# Extraction of sensing data for desired scent impressions using mass spectra of odorant molecules

**DOI:** 10.1038/s41598-022-20388-0

**Published:** 2022-09-29

**Authors:** Tanoy Debnath, Takamichi Nakamoto

**Affiliations:** 1 Department of Information and Communications Engineering, Tokyo Institute of Technology, Tokyo, Japan; 2 Laboratory for Future Interdisciplinary Research in Science and Technology, Tokyo Institute of Technology, Tokyo, Japan

**Keywords:** Cheminformatics, Olfactory system, Computational models, Machine learning

## Abstract

Most of the olfactory perception works focused on forward prediction of odor impression, for example, given an odorant’s molecular structure parameters or the sensing data predict its odor impression. So far, mapping of mass spectrum of odorant molecules into the odor perception space (binary or continuous sensory space) has been successfully performed. However, it is difficult to predict odorant’s sensing data associated with binary odor descriptors (e.g., minty, peach, vanilla etc.). In this study, we have proposed a method to extract the corresponding sensing data (mass spectrum as sensing data) for a desired scent impression although one-to-one relationships are not usually guaranteed. Our target is to extract the sensing data for a given odor descriptor that will help perfumers to create scent. This study is first report for predicting sensing data for a given binary odor descriptor.

## Introduction

Olfaction and taste are responsible for the chemical perception and recognition and the sense of smell plays an important role in perceiving the smell of good or bad things for maintaining our daily life. Odor perception is crucial for controlling the quality of food and beverages, cosmetics, industrial products, indoor air, and water pollution. We have seen a significant advancement in deep learning for vision, hearing, speech^1^, however, olfactory perception is still a progressing field for establishing quantitative structure odor relationships. When the odorant receptor (OR) is activated by an odorous substance, an electrical signal is triggered in the olfactory receptor cell and is sent to the brain through a neural process^2,3^. Odor perception is a process of combinatorial coding^4^ in the higher brain structures as one odorant can be activated by different ORs. So, an OR can respond to different olfactory stimuli that have similar smell impressions. The sensory data of different odorant molecules can be mapped onto the same type of smell impression.

Previous research has reported on the distribution of odor and odor impressions of perceptual space to estimate the minimal number of dimensions required to fully identify an olfactory perception space^5–7^. These works did not consider the sensing data similarity onto the perceptual space. To predict the perceptual quality of smell, previous attempts made significant progress using molecular structure parameters^8^, molecule’s graph structure^9^, olfactory bulb maps^10^ and other factors. Previously we utilized mass spectrometry data^11–13^ to predict the odor impressions. However, the tasks mentioned above were dedicated to solving the problem of forward prediction i.e., the prediction of scent impression from sensing data. As these works have achieved a certain level, we tried to solve the prediction of sensing data (in our case mass spectrum) from scent impression. But how can we predict the sensing data or olfactory stimuli for a given scent impression? By thinking this question, our group proposed an inverse method^14^ to predict the mass spectrum (as a sensing data) using continuous sensory information (DREAM dataset for continuous sensory information) of odor descriptors. Experimental results accurately obtain the mass spectrum feature for odor descriptors scores. However, this study did not consider binary odor descriptors (e.g., 'pine', 'minty') to predict sensing data (mass spectrum) which is more complicated because each odor molecule contains multiple odor descriptors. It is difficult to predict odorant’s sensing data associated with binary odor descriptors (e.g., minty, peach, vanilla etc.) because we need to find the sensing data similarity based on an intended scent impression. So simple clustering analysis cannot project the sensory information.

The proposed research for automatically extracting scents based on sensing data will help the perfumer to enhance or create a particular impression of the scent for a given set of fragrance descriptors. Generally, it is very difficult for a person to create the intended scent since sophisticated professional skill is required. It takes a lot of time and money for human experimentation to find perceptual similarities of same scent impressions from enormous flavor molecule databases. If we would have an automated method to predict sensing data from scent impression, we could create the actual scent based on sensing data by blending several known scents. Although we should use the mixture for that purpose, we aim to confirm whether the sensing data associated with scent impression can be found at the current stage.

In this study, we solve the inverse problem to extract the mass spectrum of similar molecules for a desired scent impression (e.g., peach, vanilla etc.). One odorant molecule can be described with several smell impressions, so several sensing data (mass spectrum) can be extracted for a given scent impression. We proposed a method to extract sensing feature space using binary odor impression utilizing compressed mass spectrometry dataset followed by a competitive learning approach called Self-organizing map (SOM)^15^. Similarity of sensing information of odorant molecules can be done by hierarchical clustering model also, however, we cannot project the sensory information at that time. We chose SOM because it provides a topology preserving mapping from the high dimensional mass spectrum space to a two-dimensional plane, as well as we can project the desired scent impression, thus extracting the sensing data for that odor descriptor.

## Materials and method

### Data

We used mass spectrum as sensing data because it can be used for both single odorant chemical and chemical mixtures^16^. Mass spectrum is considered for its ability to ionize pure molecules or complex mixtures as m/z (mass to charge ratio) at multiple fragment ion peaks with molecular ion peaks. We used the mass spectra of 2345 odorant molecules from the NIST^17^ chemistry WebBook database as a physicochemical data. We used the 51–262 m/z region of the mass spectrum as odorless molecules (intensities of m/z below 50 are mainly derived from odorless molecules such as oxygen, nitrogen) are dominant at lower m/z region and the m/z region higher than 262 has a slight contribution to odor perception for its low volatility. The odorant molecules used in this study are listed in Supplementary File 1.

Binary sensory data (e.g., peach, minty, vanilla etc.) of each odorant molecule were collected from the commercially available Leffingwell flavor database^18^ where each flavor molecule is described with several different smell impressions. In this work, we did not consider familiar odor impressions such as ‘sweet’, ‘fruity’ as these smell impressions cover a variety of other odor descriptions. On the other hand, strawberry, berry, grape etc. can be considered in the group of fruity like odor descriptions. The reason we did not use these common smell impressions is that if we want to extract this smell impression from the sensing data space, there will be a chance to get mass spectra of too many molecules.

### Method to predict mass spectrum for desired scent impression

**Figure 1:**
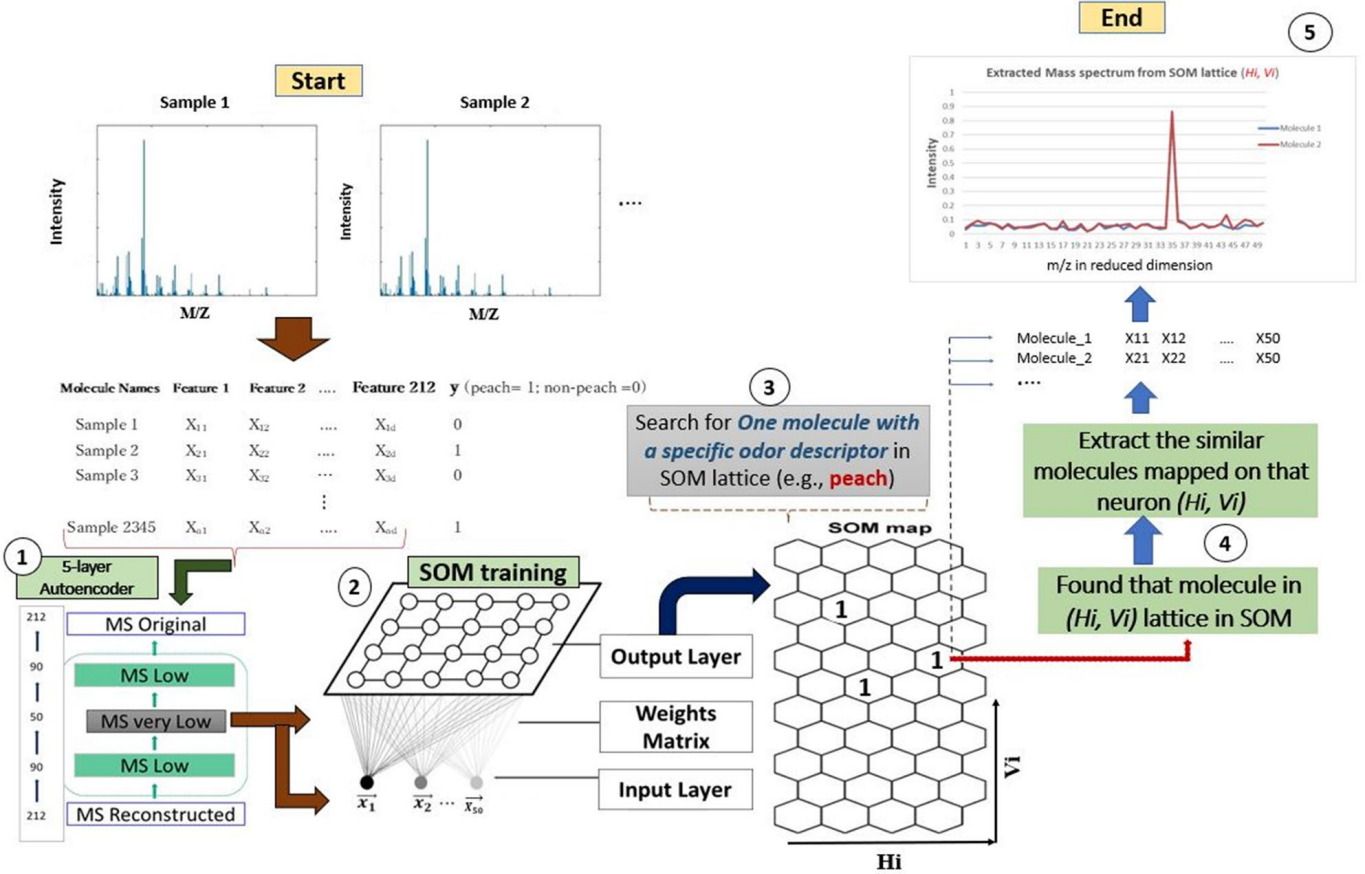
Method to extract sensing data (mass spectrum) for a desired scent impression.

We have successfully mapped the mass spectrum to binary sensory data as mentioned in the introduction. Since different mass spectra tend to cluster at the same point, we try to reverse mapping this similar mass spectrometry data for a desired odor impression. The overall procedure is illustrated in Fig. 1. We use the mass spectrum as an input feature and binary odor descriptors from the Leffingwell flavor database^17^ are used to label the mass spectrum space. First, the mass spectrum data has been compressed into lower dimensional data. Self-organized maps (SOMs) have been prepared using the lower dimensional data and an odor descriptor has been written on the lattice. When a set of odor descriptors is specified, we can get the corresponding mass spectrum from the mass spectrum space of SOM neurons.

The overall workflow is described below:

1. 2345 mass spectrum data can be compressed into lower dimension (reduced to 50 from 212 dimension optimally) using the 5-layer auto-encoder.
2. Maps are created using low-dimensional data and odor descriptors (for example, 'peach', 'spicy') are written on SOM lattice.
3. Then, we can search for the best matching unit (BMU) for odorant molecules specified with odor descriptors (for example, peach, pineapple, strawberry etc.).
4. When we find the desired molecule in (Hi = Horizontal axis, Vi = Vertical axis of SOM) lattice, we can extract the similar MS mapped on that neuron of the SOM.
5. It is possible to find three or more MS associated with the desired odor descriptor mapped in SOM’s MS space. Dimensions of these extracted mass spectra are the same as the reduced mass-spectrum from the auto-encoder since we used them for clustering the similar kinds of odorant molecules.

### Experimental procedure

Auto-encoder, a non-linear dimensional reduction method, was used to compress high dimensional mass spectrometry dataset into the latent space of the five-layer architecture. We used fivefold cross-validation to minimize the error for reproducing the original mass spectrum of odorant molecules. While fourfold was used for training the auto-encoder model, the remaining fold was used for evaluating the model. We used the mean reconstruction error of each testing fold for evaluating the 5-layer auto-encoder model. A training set of 212-dimensional m/z (vectors) (${x}_{1},{x}_{2},\dots \dots \dots .{x}_{212}$) was used as an input. A 5 layer autoencoder with l hidden layers then calculates the kth output of the nth sample, $({\mathrm{y}}_{\mathrm{kn}})$ and updates the parameters to reduce the error function E in Eq. (1), where, *λ* is the coefficient for the L2 regularization term to avoid overfitting, *β* is the coefficient of sparsity regularization term and ${\mathrm{w}}_{\mathrm{ji}}^{(\mathrm{l})}$ is the weight matrix of *i*th row of jth training example in the *l*th layer in the network including the biases. It is possible to propagate the sparsity of an auto-encoder by adding ${\Omega }_{\mathrm{sparsity}}$ to the cost function^19^. Sparsity regularizer at Eq. (1) attempts to impose a constraint on output sparsity from the hidden layer. The sigmoid activation function was used at the hidden layer of the auto-encoder. Optimization results have been reported in Table 1a.

1 $$E = \frac{1}{N}\mathop \sum \limits_{n = 1}^{N} \mathop \sum \limits_{k = 1}^{K} \left( {y_{kn} - x_{kn} } \right)^{2} + \lambda *\frac{1}{2}\mathop \sum \limits_{l} \mathop \sum \limits_{j} \mathop \sum \limits_{i} (W_{ji}^{\left( l \right)} )^{2} + \beta *\Omega_{{{\text{sparsity}}}}$$

**Table 1:** Hyperparameters used in this study. (a) Hyperparameter for optimizing the 5-layer auto-encoder, (b) condition for SOM training. SOM map size was selected based on quantization error (QE) reported in supplementary file 2 (Table s1).

Hyper-parameter	Range of Value	Optimal value
**(a)**		
Hidden layer 1	20–100	90
Hidden layer 2	5–60	50
$\eta$	0.1–0.001	0.01
L2 regularization $\lambda$	0.1–0.0001	0.0001
Sparsity regularization (*β*)	1–2	1
# Epochs in		
MS auto-encoder training		1000
# Epochs in		
Mapping network training		1000
**(b)**		
Map Size		30*20
Topological neighborhood		Hexagonal
Type of neighborhood function		Gaussian
Neighborhood Radius		1
Number of Iteration		500
Learning rate		0.5

Then we fed the reduced mass spectrometry data to the Self-organizing map. SOM is a type of Artificial Neural Network capable of transforming complex, nonlinear statistical relationships between high-dimensional data items into a low-dimensional display using the geometric relationships. In SOM analysis, each sample is considered as an n-dimensional input vector defined by its variable. An input vector is fed by an input layer in a neural network, and each is connected to a single weight output vector.

An input vector (m/z) of SOM $\overrightarrow{X}$ = (${x}_{1},\dots \dots \dots .{x}_{50}$)^*T*^*,* vector with reduced dimension from the auto-encoder, is connected to all neurons in parallel. 2345 samples with 50 dimensional reduced features were used for training the SOM in an unsupervised way. The conditions of the SOM are summarized in Table 1b. A data from the dataset is randomly selected and the distance between the node and the sample vector is calculated. The sample with the shortest Euclidean distances is considered the best matching unit (BMU) which is also called the winner node and we present the U-matrix as a hexagonal map. We expect that the similar mass spectra of the different molecules are located together at BMU of SOM. Thus, a single neuron can be the BMU for multiple data points or molecules, therefore, the number of BMUs may not be equal to the number of samples in the present cases. We used MiniSom^20^ which is a NumPy based implementation of the self-organizing map.

Our main goal is to extract the similar molecules in the SOM’s mass spectrum space. After finding the winner position for the sample, we labeled the SOM map using the desired smell impression (e.g., ‘peach’). We then searched for a specific molecule with the intended scent. After locating the winner position of that molecule in the (${H}_{i}, {V}_{i}$) neuron in the SOM, we did inverse mapping of that neuron. Inverse mapping gave us vectors of molecules whose BMU was the same for that lattice. Thus, we can extract the similar sensing data from the mass spectrum space for a specific scent impression. However, it is not certain that we will get the accurate sensing data for that desired odor impression because an odorant molecule can be expressed with several odor descriptors and a small change in the molecular structure has different odor descriptors.

In our experiment we created a head-to-tail plot of two extracted mass spectra and calculated a similarity score using R software package (OrgMassSpecR)^21^. The mass spectral similarity score is cosθ where θ is the angle between the two mass spectrum intensity vectors of two molecules. The top spectrum is used as the reference molecule and the bottom mass spectrum was the extracted molecules.

## Results and discussion

SOMs are used to map the mass spectrum of odorant molecules in binary odor impressions and clustering are shown using U-matrix representation. Quantization error (QE), a statistical metric that compares the difference between data and the results obtained by learning data from a self-organizing neural network, was calculated in the form of different map sizes (Supplementary file 2). There is a tradeoff between SOM size and the no. of molecule cluster on a SOM’s neuron. For a smaller SOM size, we expect to extract many sensing data for an odor impression. Based on the minimum QE and trying to avoid a lot of blank lattices, we chose the most appropriate map size of 600 (30*20) units. Here, we have shown several examples (using different odor descriptors) to extract the sensing data of the desired scent impression.

## Extract mass spectrum data for single odor descriptor

**Figure 2:**
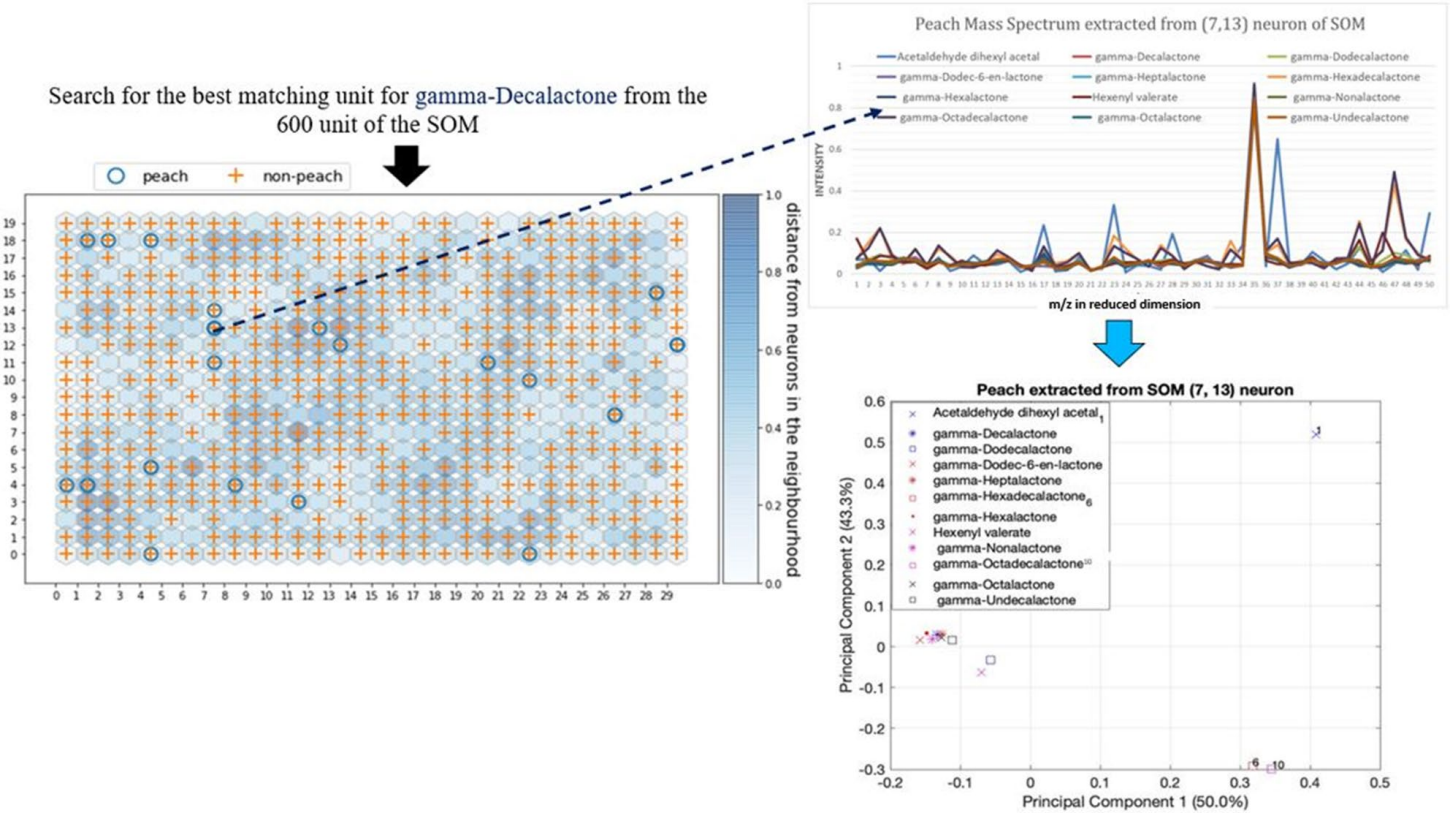
[Left side] Peach SOM (30*20) map. [Right side] Extract mass spectrum for ‘peach’ odor descriptor followed by Principal component analysis to check the closeness of the extracted molecules. Extracted mass spectrum dimension is same as the reduced dimension of mass spectrum from the Auto-encoder model. Horizontal axis values (1 to 50) represent the 50-dimensional m/z values from the bottleneck layer of the 5-layer auto-encoder.

In our experiment, 29 of the 2345 samples contained peach odor descriptors. At first Peach odor descriptor was written on SOM lattice. We searched BMU for gamma-decalactone (we chose this molecule as lactone functional group smell like peach scent impression) molecule from 600 units of SOM and found that molecule in (7,13) unit of the SOM grid as shown in Fig. 2. Then, we extracted the corresponding MS from that neuron of SOM where the similar MS were clustered. 12 mass spectra were extracted from SOM’s (7,13), including our search molecule (gamma-decalactone), i.e., 12-molecules were mapped to SOM’s neuron where BMU was the same. Principal component analysis as shown in Fig. 2 was used to test the closeness of the extracted MS of those 12 molecules where 9 out of 12 molecules were very close to each other. All the extracted molecules except acetaldehyde dihexyl acetal belong to lactone functional group, and their odor descriptors overlap with each other.

**Figure 3:**
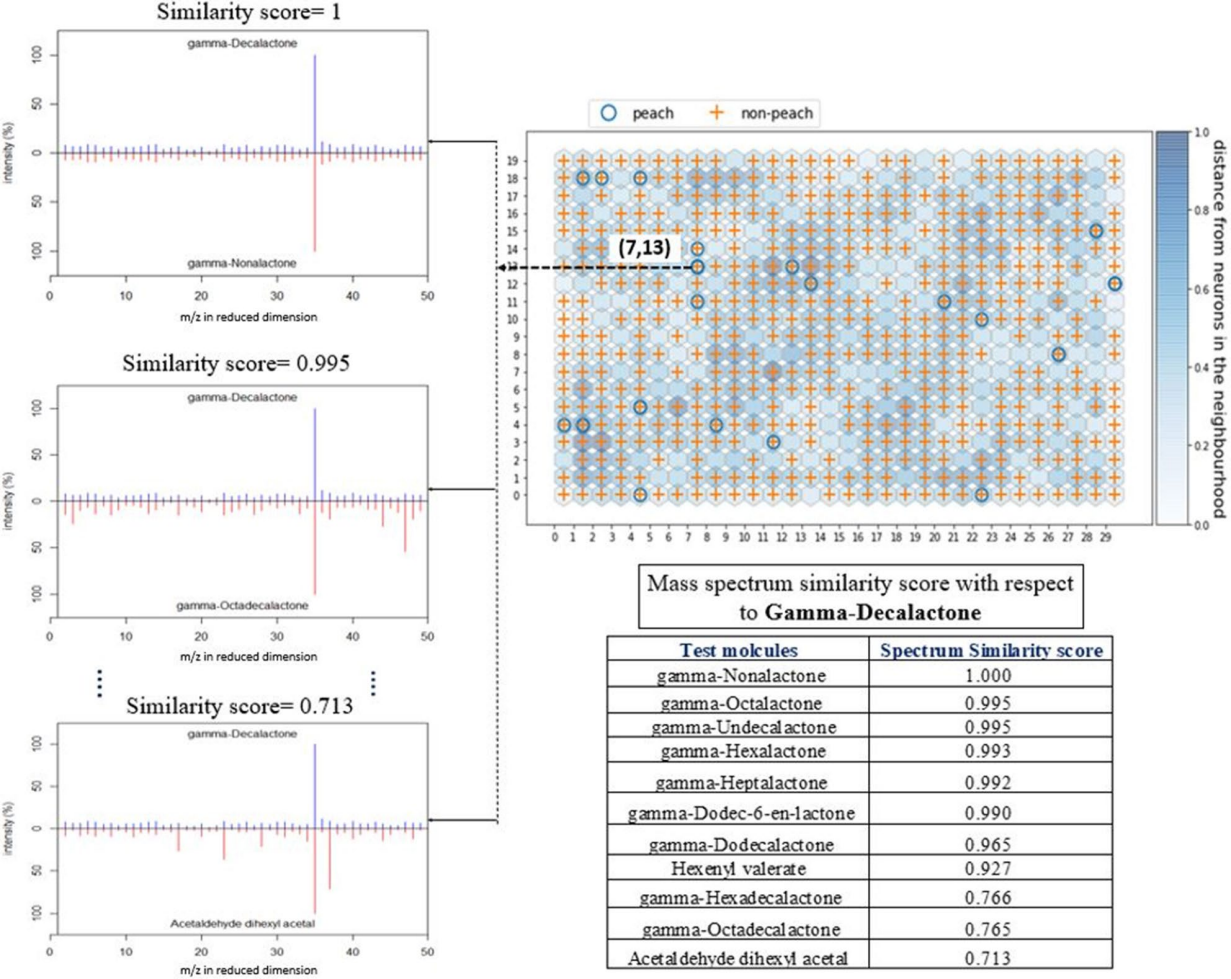
Mass spectrum similarity of the extracted molecules with respect to gamma-decalactone from the (7,13) unit of the SOM.

We also checked the mass spectrum similarity of these extracted molecules with respect to gamma-decalactone (Fig. 3) and found that their spectral similarity score is greater than 0.7. For example, mass spectrum (reduced dimension from the auto-encoder that we extracted from the SOM neuron) of gamma-nonalactone was compared with the mass spectrum of gamma-decalactone (reference molecule) and as their mass spectrum patterns are very similar, cosine similarity of these two molecules is 1. Usually, lactones are common in milk products, and they smell like peach or coconut^21^. To understand the smell impressions of these extracted molecules, we calculated the word similarity for peach like odor descriptors using Natural language processing^12^. Cosine-similarity of these odor descriptors has been shown into a two-dimensional principal component space. Similar kind of odor descriptors such as peach, pear, coconut, butter, pineapple appears closely compared to coumarin and fatty as shown in Supplementary file 2 (Fig. s1). This analysis confirmed the literature^22^ of extracted molecules of lactone functional group although word similarity was based on the down streaming task on trained English Wikipedia corpus which is not dedicated to olfactory domain. For a more concise proof, we calculated the Tanimoto similarity score for these 12 extracted molecules based on the Simplified molecular-input line-entry system (SMILES) that confirms the molecular structure similarity among these extracted molecules (Supplementary file 2 (Fig. s2)). Although we did not use molecular structure parameters in this study, we found that the odorant molecules extracted from the SOM’s (7,13) neuron for peach odor impression had high tanimoto similarity scores. Cheminformatic tool (RDKit)^23^ was used to calculate the molecular structure similarity.

## Extract mass spectrum data for two scent impressions

**Figure 4:**
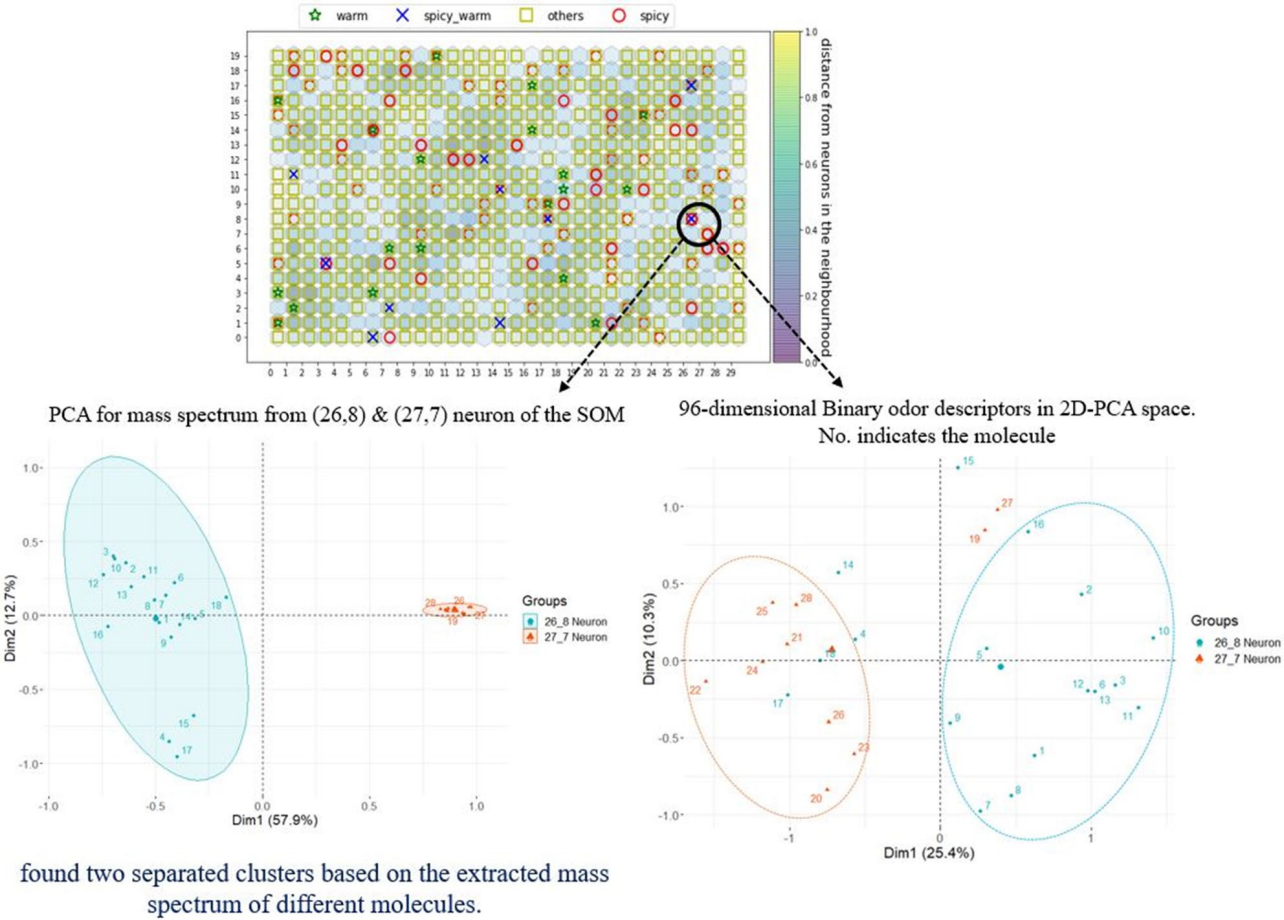
Molecules extracted from the two nearest neuron (26,8) and (27,7) of the SOM. (left side fig.) depicts the PCA for mass spectrum, (right side fig.) describes the 96-dimensional binary odor descriptors of these two neurons. Each no. indicates the odorant molecule reported in supplementary file 2 (Table S2).

We followed the same procedure described above for other odor descriptors, but with two odor descriptors. Although many molecules have the impression of specified odor descriptor, the range of molecule can be narrowed down when we use two odor descriptors. In this example, we mapped spicy and warm odor descriptors into the SOM’s mass spectrum space. For this experiment, there are 136 spicy, 19 warm and 15 samples that have both spicy and warm descriptors. We found several neurons in SOM’s 600 units that are both spicy and warm. We randomly chose a neuron of the SOM where both spicy and warm odor descriptors are present. We extracted the mass spectrum from the two nearest points of SOM, one from (26,8) neuron (both spicy and warm) and the other (27,7) neuron (only spicy smell impression). Subsequently, principal component analysis was used over these extracted molecule’s mass spectrum and two separate clusters were found based on the extracted mass spectrum of different molecules (Fig. 4-left side).

In our original database, we have a list of total 96 odor descriptors for 2345 samples. We used those to create an olfactory perceptual space for the extracted molecules even if we just used two scent impressions for extracting those molecules. The non-linearity was observed in the 96-dimensional binary sensory space (Fig. 4-right side shows the PCA of the 96-dimensional binary sensory space) in contrast to space of mass spectrum shown in the Fig. 4-left side. We found some overlapping between odor descriptors spaces for the extracted molecules from the (26,8) and (27,7) although we found some tendency to make clusters based on the molecules as a function of these two separate neurons. It is expected to have some overlapping because one molecule can be described with several smell impressions. Although we only mapped the spicy and warm odor descriptors in the mass spectrum space of the SOM, we checked the tendency of the extracted molecule’s sensory space with their respective odor impressions (all smell impressions) from the Leffingwell database. It is clear from the (Fig. 4-right side) that even if we only use our desired smell impression (like spicy or warm), we can extract the related molecules from the sensing data space and these extracted molecules have sufficient accuracy in the binary odor descriptors spaces.

To find a common characteristic, we did the same experiment with other neuron of the SOM where spicy and warm clustered together and the nearest neuron with only spicy odor impression. As we know that eugenol gives warm, spicy scent^22^, we find out the location of this molecule in the (17,8) neuron of the SOM and extracted the mass spectrum clustered on that neuron. This time we extracted the molecules from the (17,8) neuron of the SOM where both spicy and warm odor descriptors were clustered and from the nearest (18,7) neuron. We found the same tendency as above. Mass spectrum similarity is also calculated using eugenol (Odor descriptor: spicy, warm, pungent, smoky) as a reference for the (17,8) lattice in Supplementary file 2 (Fig. s3). We found high cosine similarity of each molecule’s mass spectrum except cinnamaldehyde although extracted molecule’s odor descriptors are correlated with each other. We reported a set of odor descriptors of those extracted molecules in Table S3.

We also performed the similar kind of experiment with different pair, for example, ‘Cooling and minty’, ‘strawberry and pineapple’ depicted in Supplementary file 2 (Figs. s4 and s5 respectively). For each of these experiments we extracted the mass spectrum from the SOM neuron where each pair of odor descriptors (e.g., cooling and minty) located in the same lattice of the SOM and from the nearest neuron with only one odor descriptor. Duo trio sensory test was performed to check whether the same neuron molecules smell alike or not. In the next section we will describe the sensory evaluation of the extracted flavor molecules from the same SOM grid compared with the nearest neuron.

### Sensory evaluation of the extracted flavor molecules

We performed Duo trio sensory test to see whether two molecules extracted from the same neuron had the same odor impression or not. Here, a subject was asked to pick up either of two samples closer to the reference one. First, the subject sniffed the reference sample and needed to remember the smell. Then, the two samples were given, and the subject needed to choose which one was closer to the reference one. The one sample was based on the molecule extracted from the SOM grid and another one was from the nearest neuron of the SOM. The original samples (30 μl) were diluted with ethanol (270 μl) and presented to the non-expert participants (total 21 in this experiment; age between 21 and 40). Each participant had clear nose during the experiment and all participants provided informed consent in the experiment sheet. The sensory test was done under the approval by the human subject research ethics review committee of Tokyo Institute of Technology in accordance with Helsinki declaration (Approval Number. 2020287). Furthermore, result was calculated using one proportion binomial test (right tailed) since our sample size is small.

Out of 21 participants 16 people reported that eugenyl acetate was close to the reference sample (cinnamaldehyde) (odor descriptors: sweet, spicy, warm, cinnamon) [both are from the (17,8) neuron of the SOM] in Table 2. Since *p* value < *α* = 0.05, null hypothesis was binomially distributed can be rejected. So, eugenyl acetate was much closer to the reference sample than methyl isoeugenol (from^7,18^ neuron of SOM).

**Table 2:** Duo trio sensory test result between eugenyl acetate (17,8 neuron) and methyl isoeugenol (18,7 neuron)-cinnamaldehyde (17,8 neuron) as a reference sample.

Eugenyl acetate (Fruity, sweet, spicy, balsamic, warm)	Methyl isoeugenol (Sweet, floral, spicy, tea)	Z statistic	*P* Value
16	5	2.38	0.0133018

We also got the same observation for the cooling and minty cases where the cis-3,3,5-trimethylcyclohexan-1-ol was much closer to the reference sample- menthol (odor descriptors: cooling, minty, mentholic) [both are from different neuron of the SOM] and there was a statistically significant difference between cis-3,3,5-trimethylcyclohexan-1-ol and 2,4-lutidine although they are close in MS space as shown in Fig. S4 and Table 3. Note that, 2,4-lutidine has phenolic and smoky odor descriptors that distinguishes this flavor molecule from the reference sample. Although menthol was from different neuron of SOM, odor descriptors of these two molecules overlaps (cooling and minty). The most important point in this experiment is that although the structure of the odorant molecules is different, their odor descriptors match. So, we can alternatively use any one of these molecules. This will be clear in the next experiment of Table 4 where we have chosen a reference molecule which has the similar smell impressions for both molecules with a different odor descriptor (roast) for strawberry furanone.

**Table 3:** Duo trio sensory test result between cis-3,3,5-Trimethylcyclohexan-1-ol (18,9 neuron) and methyl isoeugenol (17,9 neuron)- menthol (29,19 neuron) as a reference sample.

Cis-3,3,5-Trimethylcyclohexan-1-ol (Herbal, minty, camphoraceous, cooling)	2,4-Lutidine (Green, minty, phenolic, fishy, smoky)	Z -statistic	P Value
20	1	4.12	1.04904E-05

**Table 4:** Duo trio sensory test result between strawberry furanone (14,19 neuron) and methyl 3-hexenoate (14,18 neuron)- ethyl hexanoate (10,12 neuron) as a reference sample.

Strawberry furanone (Fruity, pineapple, roast, strawberry)	Methyl 3-hexenoate (Fruity, green, tropical, pineapple, honey)	Z statistic	*P* Value
4	17	− 2.84	0.999

We used the ethyl-hexanoate (odor descriptors: fruity, tropical, pineapple, pear, banana, strawberry) that was not from the SOM’s (14,19) neuron. Only 4 among 21 participants reported that strawberry furanone (from 14,19 neuron of the SOM) was close to the referred sample (ethyl hexanoate) in Table 4. Roast odor descriptors of furanone affects the sensory perception of participants towards the methyl 3-hexenoate although strawberry furanone and ethyl-hexanoate have both pineapple and strawberry scent impressions in common. One hypothesis that describes this trend can be described with semantic similarity reported in Supplementary file (Fig. S1-b). We found that roast odor descriptors describe different odor impression than pineapple and strawberry that is unpleasant to the participants while doing the sensory test. Notice that, pineapple, strawberry, honey is close to each other (Fig. S1-b) based on the word similarity, however, the distance between roast and (pineapple, strawberry) odor descriptors are not too close. So, it is important to have a similar kind of odor descriptors while choosing the similar kind of flavor molecules.

Above results confirmed that the extracted molecules located in the same neuron have the same perceptual similarity. Even if we did not consider odor descriptor while clustering the mass spectrum of the odorant molecules, we got the similar odorant molecules based on their spectrum similarity. Moreover, if we chose two different molecules with almost similar odor descriptors with a different (‘fishy’ or ‘roast’ in this experiment) smell impression, it would affect the alternation of the intended scent. So, both sensing data and odor descriptors similarity need to be considered when creating the odors.

## Conclusion

In this study, we proposed a mathematical model to extract the sensing data for an intended binary odor descriptor without continuous sensory information. This research is completely different from the previous study that used forward perceptual model to map the physicochemical properties of odorant molecules to the binary or continuous sensory data. This is the very first attempt to extract sensing data automatically for a desired scent impression that will be helpful to create scents.

This proposed model (model to predict chemical compound from odor descriptors) that has been developed by the method of dimension reduction following competitive learning methods not only clusters similar odor molecules based on their mass spectrum similarity but also identifies the desired molecules with a specific odor descriptor. If we add a new potential odorant compound to the database where the odor descriptor is unknown, we can cluster that compound in the sensing data space and we can select the odor descriptor based on sensing data similarity to compounds with known label, which is the closest to new odorant. Experimental results show that multiple mass spectra associated with a given odor descriptor can be extracted and we have confirmed the extracted mass spectrum similarity. Moreover, we also did the duo trio sensory test to evaluate the scent similarity extracted from the same neuron of the SOM and result shows that molecules located in the same neuron have the same perceptual similarity compared to the nearest neuron of the SOM. We also showed that odor descriptors play an important role for choosing the intended molecule for a desired scent. We found that even if we did not give all input information of binary odor descriptors, we still got the mass spectrum of similar flavor molecules. This study is the first demonstration for the smart olfactory display where we can generate the scent by blending different odor components for different combination of desired scent impression.

Potential application of this research can be in the perfume or food industries who tries to find the structural similarity of odorant molecules for a particular scent impression. This will help scientists to automatically get the similar flavor molecules for a desired scent impression without manual operation. Our current work was based on single molecules only, but this needs to be proven with chemical mixtures. This work can be extended to chemical mixtures because the odor we smell is a complex mixture of multiple monomolecular components.

## Supplementary Information

Supplementary Information 1.

Supplementary Information 2.

## Data Availability

The dataset used and/or analysed during the current study are not publicly available (as it is a commercial database) but are available from the (Corresponding Author: Takamichi Nakamoto) on reasonable request.

## References

[1] Lecun Y, Bengio Y, Hinton G (2015). Deep learning. Nature.

[2] Buck L, Axel R (1991). A novel multigene family may encode odorant receptors: A molecular basis for odor recognition. Cell.

[3] Nakamoto T (2016). Essentials of Machine Olfaction and Taste.

[4] Malnic B, Hirono J, Sato T, Buck L (1999). Combinatorial receptor codes for odors. Cell.

[5] Castro JB, Ramanathan A, Chennubhotla CS (2013). Categorical dimensions of human odor descriptor space revealed by non-negative matrix factorization. PLoS ONE.

[6] Koulakov AA, Kolterman BE, Enikolopov AG, Rinberg D (2011). In search of the structure of human olfactory space. Front. Syst. Neurosci..

[7] Madany Mamlouk A, Chee-Ruiter C, Hofmann UG, Bower JM (2003). Quantifying olfactory perception: Mapping olfactory perception space by using multidimensional scaling and self-organizing maps. Neurocomputing.

[8] Keller A (2017). Predicting human olfactory perception from chemical features of odor molecules. Science.

[9] Sanchez-Lengeling B. et al., Machine learning for scent: Learning generalizable perceptual representations of small molecules. Preprint at https://arxiv.org/abs/1910.10685 (2019).

[10] Shang L, Liu C, Tomiura Y, Hayashi K (2018). Odorant clustering based on molecular parameter-feature extraction and imaging analysis of olfactory bulb odor maps. Sens. Actuators B Chem..

[11] Nozaki Y, Nakamoto T (2016). Odor impression prediction from mass spectra. PLoS ONE.

[12] Debnath T, Nakamoto T (2020). Predicting human odor perception represented by continuous values from mass spectra of essential oils resembling chemical mixtures. PLoS ONE.

[13] Debnath T, Nakamoto T (2022). Predicting individual perceptual scent impression from imbalanced dataset using mass spectrum of odorant molecules. Sci. Rep..

[14] Hasebe D, Alexandre M, Nakamoto T (2022). Exploration of sensing data to realize intended odor impression using mass spectrum of odor mixture. PLoS ONE.

[15] Kohonen T (1990). The self-organizing map. Proc. IEEE.

[16] Debnath T, Prasetyawan D, Nakamoto T (2021). Predicting odor perception of mixed scent from mass spectrometry. J. Electrochem. Soc..

[17] CAS Number Search. http://webbook.nist.gov/chemistry/cas-ser.html

[18] Leffingwell J. C. Leffingwell & Associates (2005).

[19] B. A. Olshausen & D. J. Fieldt. Sparse coding with an overcomplete basis set: A strategy employed by V1? Vision research, **37**(23), 3311–3325, pmid: 9425546 (1997). 10.1016/s0042-6989(97)00169-7 9425546

[20] Vettigli, G. MiniSom: Minimalistic and NumPy-based implementation of the self organizing map, https://github.com/JustGlowing/minisom/ (2018).

[21] Spectrum similarity: Similarity between two mass spectra, https://rdrr.io/rforge/OrgMassSpecR/man/SpectrumSimilarity.html

[22] Coucquyt, P., Lahousse, B., Langenbick, J. The art and science of food pairing, Octopus Publishing group Ltd., (2020).

[23] RDKit: Open-source cheminformatics. http://www.rdkit.org.

