# Supplementary material for: Extraction of sensing data for desired scent impressions using mass spectra of odorant molecules
Source: Sci Rep. 2022 Sep 29;12:16297. doi: 10.1038/s41598-022-20388-0 (PMC9522809; doi:10.1038/s41598-022-20388-0)

**Table S1:** Map size and Quantization error for different SOM size.

| Map size | 100 (10*10) | 200 (20*10) | 300 (30*10) | 400 (20*20) | 600 (30*20) | 800 (40*20) |
| --- | --- | --- | --- | --- | --- | --- |
| QE | 0.654 | 0.573 | 0.523 | 0.484 | 0.422 | 0.373 |

**Fig S1** Descriptions: We used pre-trained word vectors trained on English Wikipedia using Fast-Text. English pre-trained word vector (300 dimensions) was downloaded (6.59 GB) and there are 2519370 tokens available^1^. After that, the cosine similarity of peach like odor descriptors from the 12 extracted molecules was calculated using pre-trained English wiki Fast-Text model followed by the PCA to show the results in two-dimensional space.

Reference

1. T. Mikolov, E. Grave, P. Bojanowski, C. Puhrsch, A. Joulin. Advances in Pre-Training Distributed Word Representations, Proceedings of the International Conference on Language Resources and Evaluation (LREC 2018). https://aclanthology.org/L18-1008
2. Word Similarity of Peach odor Descriptor


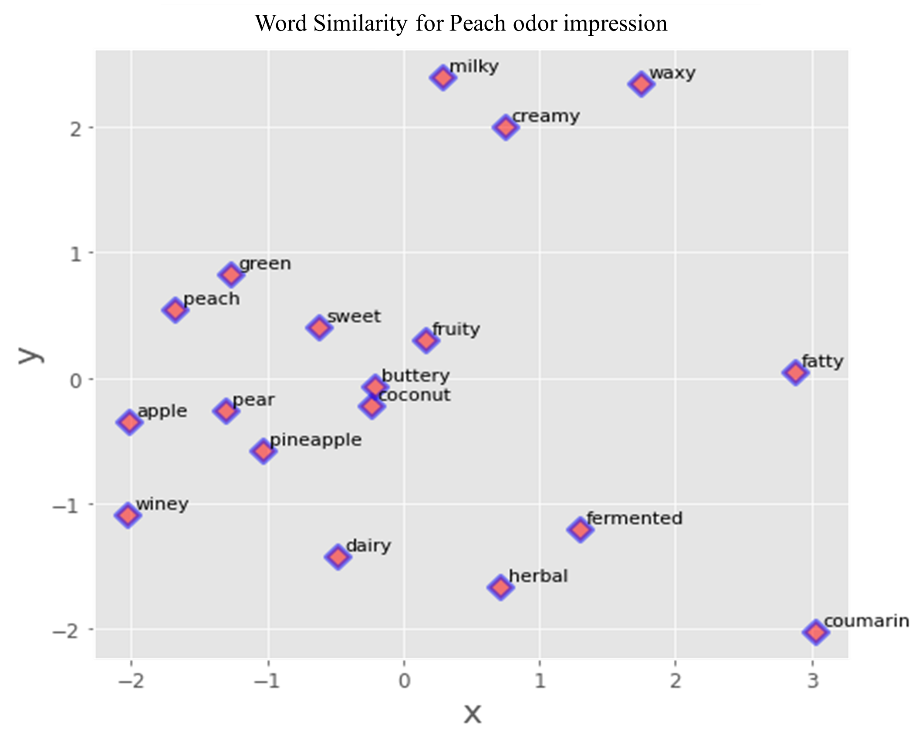


1. Word Similarity of Pineapple & strawberry odor Descriptors


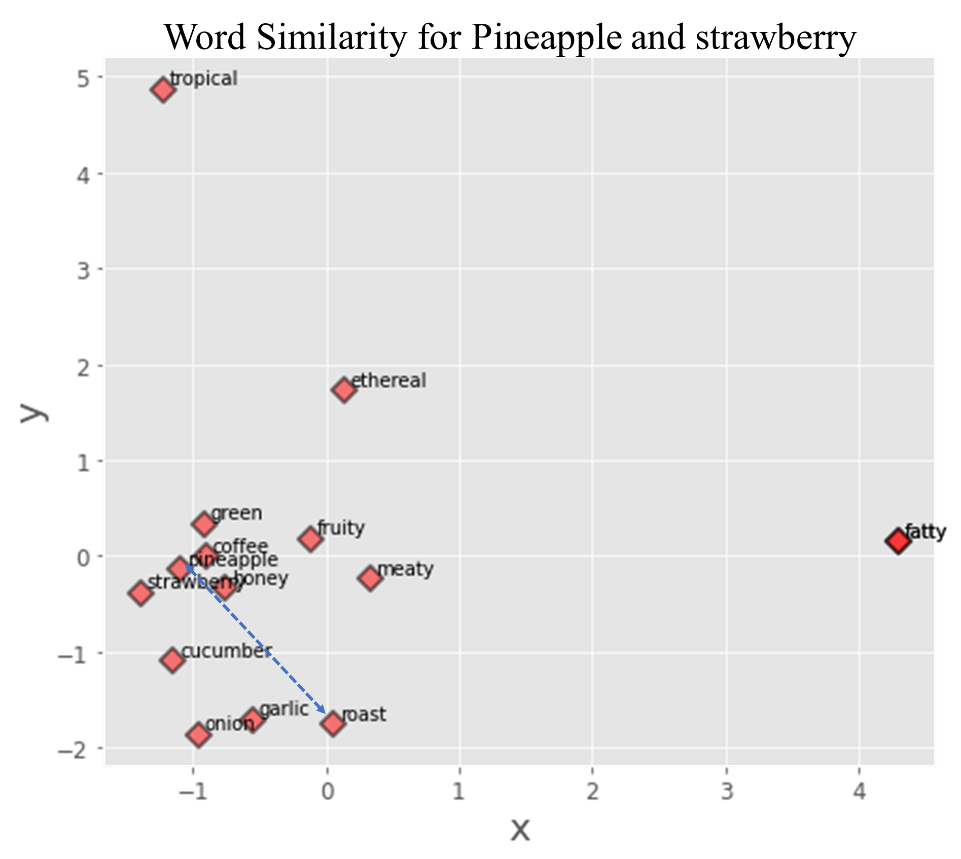


**Fig S2:** Tanimoto similarity score for the extracted molecules based on the Simplified molecular-input line-entry system (SMILES) that confirms the similarity of molecular structure similarity between these extracted molecules from the (7,13) neuron of the SOM.


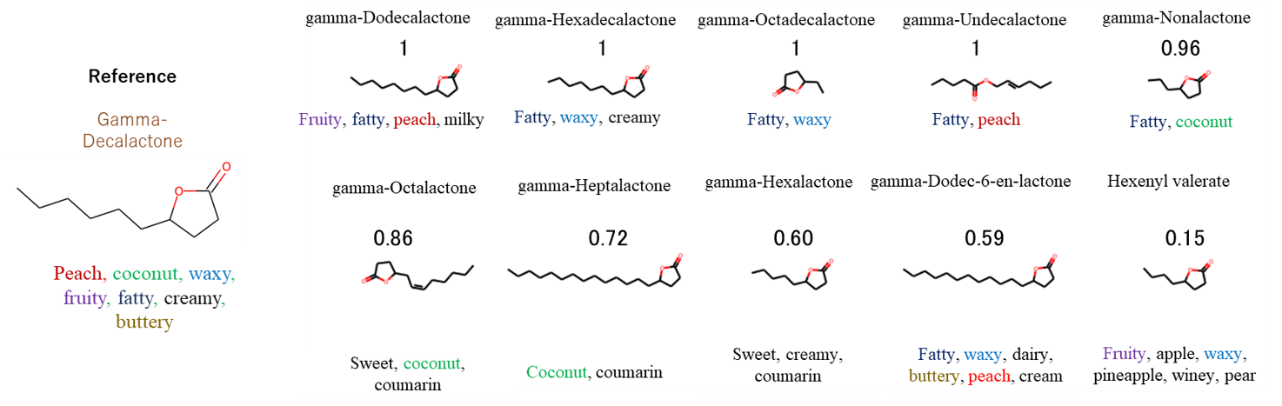


**Table S2:** Molecules extracted from the two nearest neuron (26,8) & (27,7) of the SOM. Name of the molecules with the index no. (as shown in Fig. 4 in the article) are reported below.

| **Index no.** | **Molecule extracted from the SOM neuron** | **Name of the odorant molecule** |
| --- | --- | --- |
| 1 | 26_8 Neuron | Allyl cinnamate |
| 2 | 26_8 Neuron | Amyl cinnamate; Pentyl cinnamate |
| 3 | 26_8 Neuron | Butyl cinnamate; n-Butyl cinnamate |
| 4 | 26_8 Neuron | Butylidene phthalide; 3-n-Butylidenephthalide |
| 5 | 26_8 Neuron | Cinnamyl cinnamate |
| 6 | 26_8 Neuron | Cyclohexyl cinnamate |
| 7 | 26_8 Neuron | Ethyl acetyl phenylpropionate; Ethyl benzyl acetoacetate |
| 8 | 26_8 Neuron | Ethyl cinnamate |
| 9 | 26_8 Neuron | Heptyl heptanoate |
| 10 | 26_8 Neuron | Isoamyl cinnamate; Isopentyl cinnamate |
| 11 | 26_8 Neuron | Isobutyl cinnamate |
| 12 | 26_8 Neuron | Isopropyl cinnamate |
| 13 | 26_8 Neuron | Methyl cinnamate |
| 14 | 26_8 Neuron | Methyl benzofuran; 2-Methylbenzofuran |
| 15 | 26_8 Neuron | Methyl coumarin; 6-Methylcoumarin |
| 16 | 26_8 Neuron | Phenyl butenone; 4-Phenyl-3-buten-2-one |
| 17 | 26_8 Neuron | Propylidenephthalide; 3-Propylidenephthalide |
| 18 | 26_8 Neuron | Safrole |
| 19 | 27_7 Neuron | Acetanisole; 4-Methoxyacetophenone; |
| 20 | 27_7 Neuron | Acetyl thymol; Thymol acetate |
| 21 | 27_7 Neuron | Butylphenol; 3-tert-Butylphenol |
| 22 | 27_7 Neuron | Carvacrol; 2-Methyl-5-isopropylphenol |
| 23 | 27_7 Neuron | Carvacryl acetate; 5-Isopropyl-2-methyl phenyl acetate |
| 24 | 27_7 Neuron | Dimethyl ethyl phenol; 4-(1,1-Dimethylethyl) phenol |
| 25 | 27_7 Neuron | Hydroxy methylacetophenone; 2-Hydroxy-5-methylacetophenone |
| 26 | 27_7 Neuron | Isopropyl benzyl alcohol; p-Isopropylbenzyl alcohol |
| 27 | 27_7 Neuron | Methylionone; alpha-iso-Methylionone; gamma-Methylionone |
| 28 | 27_7 Neuron | Thymol; 5-Methyl-2-isopropylphenol |

**Fig S3:** Mass spectrum similarity score for the extracted molecules from the SOM’s (17,8) neuron with respect to Eugenol (Odor descriptor: spicy, warm, pungent, smoky).


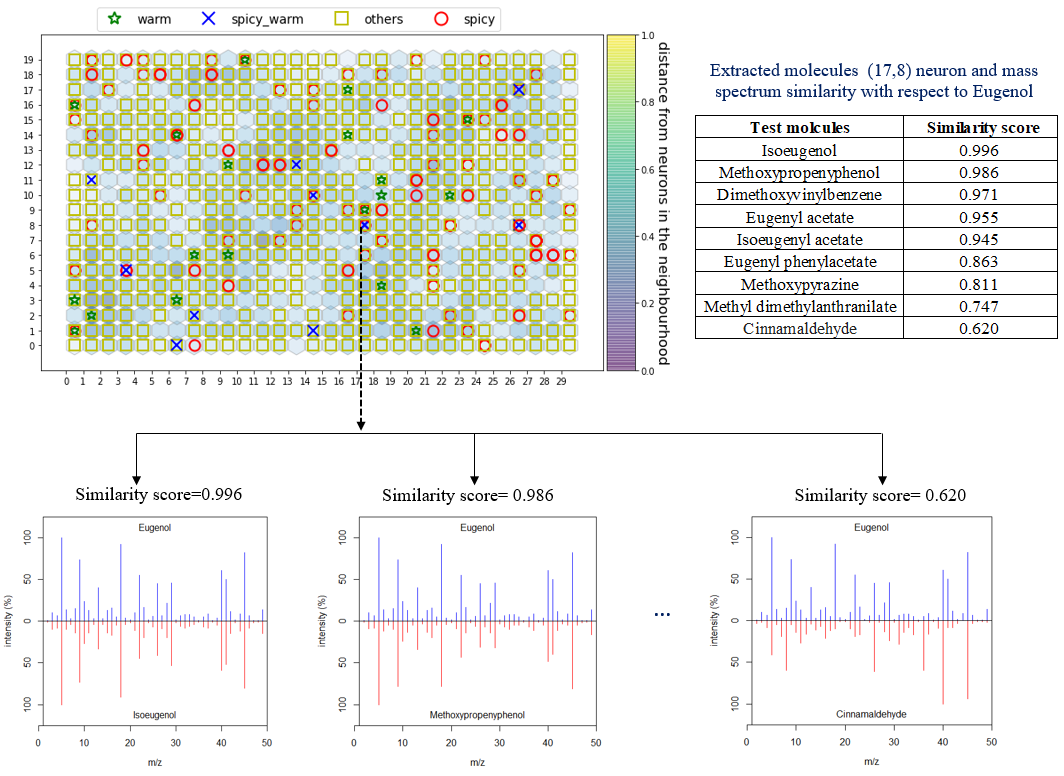


**Table S3:** Odor Descriptors of the extracted molecules from the (17,8) neuron of the SOM.

| **Test molcules** | **Similarity score** | Odor Descriptors |  |  |  |  |
| --- | --- | --- | --- | --- | --- | --- |
| Isoeugenol | 0.996 | spicy | warm |  |  |  |
| Methoxypropenyphenol | 0.986 | sweet | spicy | phenolic | smoky |  |
| Dimethoxyvinylbenzene | 0.971 | sweet | earthy | phenolic | smoky | medicinal |
| Eugenyl acetate | 0.955 | fruity | sweet | spicy | balsamic | warm |
| Isoeugenyl acetate | 0.945 | fruity | sweet | floral | spicy | balsamic |
| Eugenyl phenylacetate | 0.863 | sweet | spicy | honey | warm | aromatic |
| Methoxypyrazine | 0.811 | green | sweet | earthy | nutty | roast |
| Methyl dimethylanthranilate | 0.747 | musty | grape |  |  |  |
| Cinnamaldehyde | 0.620 | sweet | spicy | warm | cinnamon |  |

**Fig S4**. SOM of Cooling & minty odor descriptors


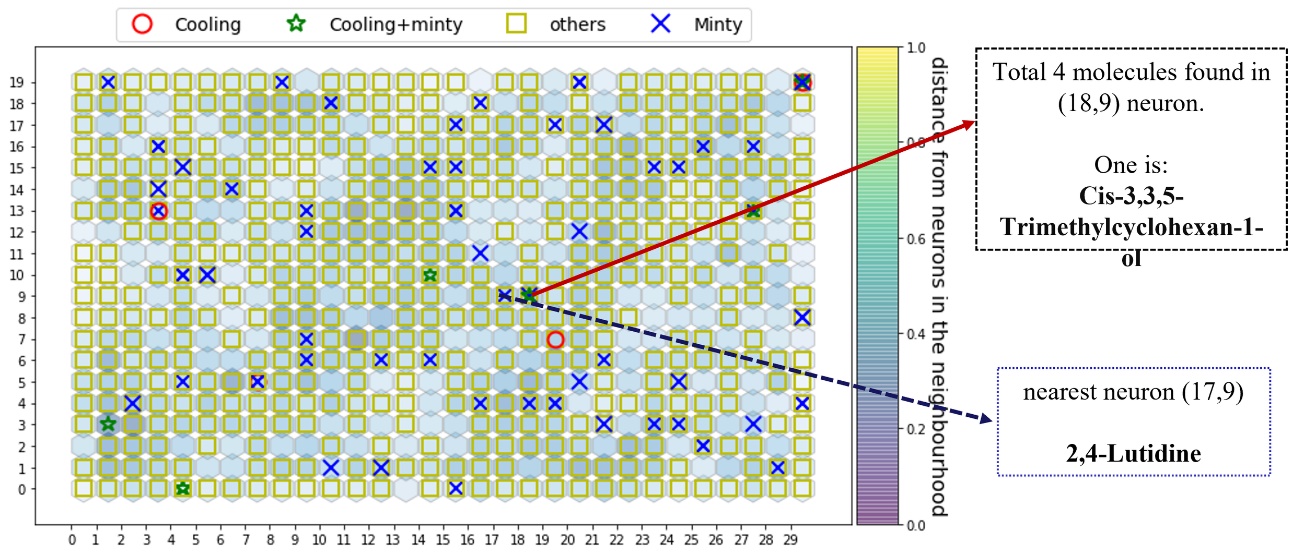


**Fig S5.** Mass spectrum similarity score for pineapple & strawberry. Two molecules extracted from the SOM’s (14,19) neuron and one of the extracted molecules from SOM’s (14,18) unit.


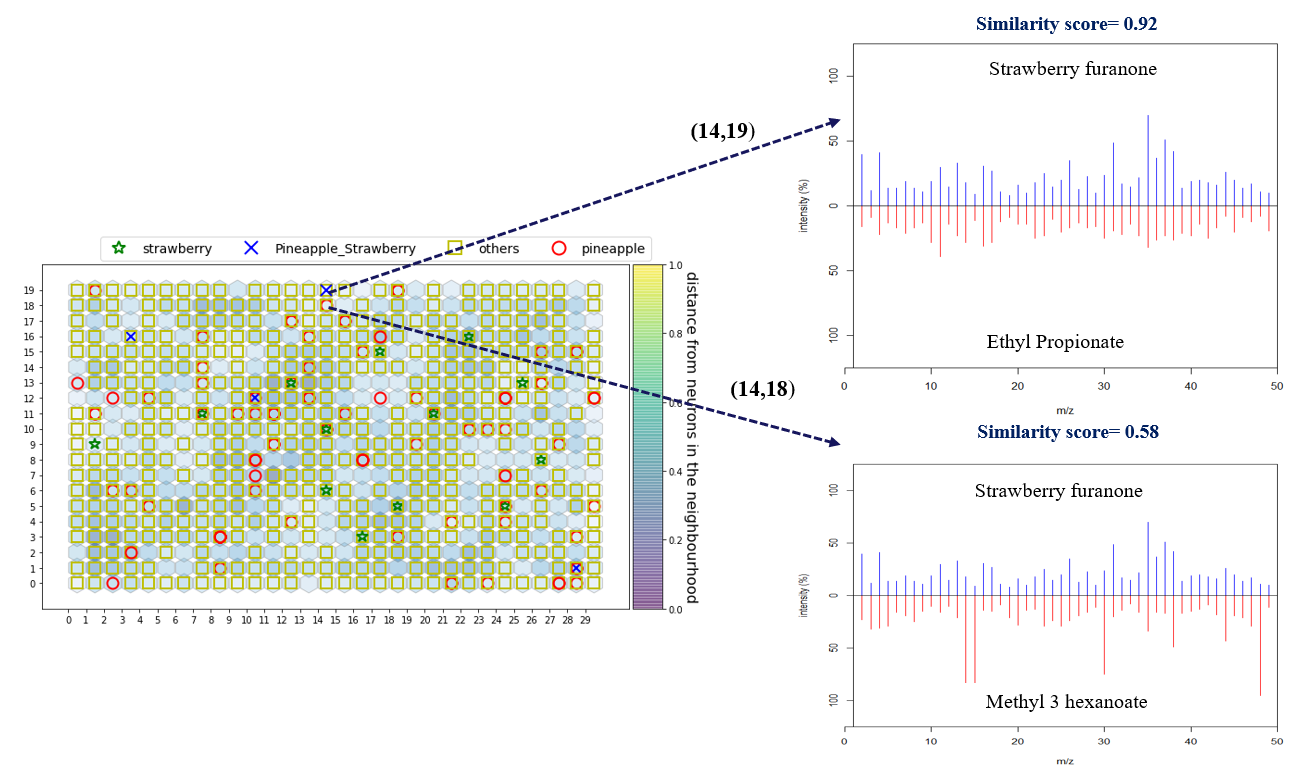

Supplement: Supplementary file 2 — Supplementary Information 2. [file 41598_2022_20388_MOESM2_ESM.docx]
